# Screening of cellulose-degrading bacteria and optimization of cellulase production from *Bacillus cereus* A49 through response surface methodology

**DOI:** 10.1038/s41598-024-58540-7

**Published:** 2024-04-02

**Authors:** Jinjun Wang, Fei Bao, Huixian Wei, Yang Zhang

**Affiliations:** 1 Key Laboratory of Arable Land Quality Monitoring and Evaluation, Ministry of Agriculture and Rural Affairs, Yangzhou University, Yangzhou, 225009 Jiangsu China; 2 Present Address: College of Environmental Science and Engineering, Yangzhou University, Yangzhou, 225127 Jiangsu China

**Keywords:** Applied microbiology, Environmental microbiology

## Abstract

Cellulose-degrading microorganisms hold immense significance in utilizing cellulose resources efficiently. The screening of natural cellulase bacteria and the optimization of fermentation conditions are the hot spots of research. This study meticulously screened cellulose-degrading bacteria from mixed soil samples adopting a multi-step approach, encompassing preliminary culture medium screening, Congo red medium-based re-screening, and quantification of cellulase activity across various strains. Particularly, three robust cellulase-producing strains were identified: A24 (MT740356.1 *Brevibacillus borstelensis*), A49 (MT740358.1 *Bacillus cereus*), and A61 (MT740357.1 *Paenibacillus* sp.). For subsequent cultivation experiments, the growth curves of the three obtained isolates were monitored diligently. Additionally, optimal CMCase production conditions were determined, keeping CMCase activity as a key metric, through a series of single-factor experiments: agitation speed, cultivation temperature, unit medium concentration, and inoculum volume. Maximum CMCase production was observed at 150 rpm/37 °C, doubling the unit medium addition, and a 5 mL inoculation volume. Further optimization was conducted using the selected isolate A49 employing response surface methodology. The software model recommended a 2.21fold unit medium addition, 36.11 °C temperature, and 4.91 mL inoculant volume for optimal CMCase production. Consequently, three parallel experiments were conducted based on predicted conditions consistently yielding an average CMCase production activity of 15.63 U/mL, closely aligning with the predicted value of 16.41 U/mL. These findings validated the reliability of the model and demonstrated the effectiveness of optimized CMCase production conditions for isolate A49.

## Introduction

Cellulose is one of the most abundant renewable resources currently available^1^. Photosynthesis produces over 150 million tons of cellulose annually, and the vast majority has yet to be utilized^2^, making its degradation an active research hotspot. Cellulose is a polysaccharide substance composed of β-1, 4-glucose residues^3^, and the primary component of vascular and lichen plants, along with certain algal cell walls. This stacked macromolecular chain polymer^4^, exhibits an inherently stable structure, impeding its decomposition and utilization under environmental conditions^5^. The standard cellulose-degrading methods include physical, chemical, and biodegradation^6^, of which biodegradation emerges as an optimal approach owing to its enhanced effectiveness, reduced cost, and minimal environmental impact^7^. The biodegradation method involves cellulase as the key enzyme that catalyzes the conversion of cellulose into readily available glucose^8^. This enzymatic conversion of cellulose into utilizable glucose generally encompasses the synergistic interplay of three enzymes^9^, namely endo-glucanase, exo-cellulase, and β-glucosidase^10–12^. The process of cellulose degradation is typically divided into three steps. First, the long-chain oligosaccharides are cleaved into short-chain oligosaccharides. Next, the short-chain oligosaccharides are degraded to release cellobiose, which is ultimately hydrolyzed into glucose^13^. Notably, cellulose-degrading strains differ considerably in their degradation ability, which is further influenced by varying culture conditions. The screening and cultivation of cellulose-degrading bacteria bear profound importance in the utilization of cellulose resources. In China, strains demonstrating remarkable cellulose-degrading ability were isolated from corn straw compost^14^, which were subsequently formulated into an exogenous cellulose-degrading bacteria (ECDB) microbial inoculum for introduction to the compost bin for experimental purposes. The addition of ECDB transformed the humus composition and increased the abundance of bacteria associated with humus formation, thus promoting the biological pathway of humus synthesis and improving its utilization. In a similar report, four bacterial strains exhibiting cellulose-degrading capability were successfully isolated in the green and food waste compost^15^. In comparison to compost devoid of inoculated strains, those infused with cellulose-degrading bacteria showed an improvement in the quality of compost products, which could substitute some fertilizers and in turn hold specific economic value. Numerous studies have demonstrated the notable research significance of cellulose-degrading microorganisms. Therefore, screening and cultivation of new cellulose-degrading bacteria stand as promising avenues that can advance the development of the cellulose industry and promote the sustainable utilization of cellulose resources.

*Bacillus subtilis* exhibits enormous potential as a suitable host for biochemical production, primarily due to its excellent growth characteristics and adept bioresource utilization^16^. It is often employed as a host carrier for the production of industrially important enzymes such as lysozyme, cellulase, and amylase as well as for the synthesis of specific substances, including selenium nanoparticles, and other essential compounds^17,18^. Accordingly, this study shows a keen interest in cellulase-producing *Bacillus subtilis* strains and further aims to obtain the target strain through systematic screening experiments and optimization of the cultivation conditions.

To explore the CMCase production ability of the screened isolates under varying conditions, a series of single-factor experiments were designed. Based on the results, the Box Behnken design for^19^ response surface methodology (RSM) was applied to optimize the cellulase enzyme production conditions for a specific selected strain. Hence, this optimization process could serve as a valuable reference to enhance enzyme production by cellulose-degrading bacteria.

## Material and methods

### Materials

#### Main reagents

All the reagents used in this study were purchased from Yangtai Medical Devices Co, Yangzhou, Jiangsu Province, China: agar powder, potassium dihydrogen phosphate, magnesium sulfate, gelatin, sodium carbonate, disodium hydrogen phosphate, potassium dihydrogen phosphate, potassium chloride, yeast extract powder, acid hydrolyzed casein, Congo red, hydrogen chloride, 3,5-dinitro salicylic acid (DNS) reagent, sodium carboxymethyl cellulose (CMC Na), glucose, and microcrystalline cellulose powder.

#### Culture media

Isolation and screening medium^20^: MgSO_4_ (0.25 g) and KH_2_PO_4_ (0.5 g), cellulose powder (2 g; filtered by soaking in 1 mmol/L HCl), agar (16 g), gelatin (2 g) and distilled water (1000 mL), pH 7.0.

Identifying medium^20^: Na_2_CO_3_ (1 g), Na_2_HPO_4_ (1.2 g), KH_2_PO_4_ (0.9 g), MgSO_4_ (0.5 g), KCL (0.5 g), yeast extract powder (0.5 g), acid hydrolyzed casein (0.5 g), Congo red (0.2 g), cellulose powder (5.0 g), agar (15.0 g), pH 6.9–7.1.

Liquid fermentation medium^20^: cellulose powder (2 g; filtered by soaking with 1 mmol/L HCl), gelatin (2 g), MgSO_4_-7H_2_O (0.25 g), KH_2_PO_4_ (0.5 g), distilled water (1000 mL), pH 7.0.

Preservation medium: Nutrient broth (NB) and Luria Bertani (LB).

All media were autoclaved at 121 °C for 30 min.

#### Collection and processing of mixed soil samples

Samples were collected from the soil, wormcast, pig manure, and cow dung at the straws-returned field in the vicinity of the College of Agriculture, Yangzhou University, Yangzhou, Jiangsu Province, China (32.389204° N and 119.423282° E). All the samples were weighed (5 g) and mixed into conical flasks by adding 100 mL of sterile water and shaken vigorously for 20 min. Set the stirred liquid concentration to 10^0^. Next, 10 mL of the oscillating liquid was added to 90 mL of sterile water to make a 10^−1^ bacterial suspension. Consecutively, the suspension was subjected to a series of dilutions, including 10^−2^, 10^−3^, 10^−4^, 10^−5^, 10^−6^, and 10^−7^ for further studies^21^.

### Screening of cellulose decomposing strains

#### Primary and re-screening of strains

Each of the gradient dilutions was evenly spread across the isolation and screening medium and subsequently incubated at 30 °C in an incubator for 10 days. During the incubation period, the morphological characteristics of the growing bacteria were monitored. Moreover, the better-growing strains were singled out and selected from the culture plates, and the purified isolated colonies were obtained after successive streaking cultivation on agar plates. Next, each colony was inoculated onto Congo red cellulose identification medium and cultured under identical conditions-same 30 °C in an incubator for 10 days^22^. The isolates were initially screened by measuring the size of the transparent zone on the Congo red cellulose medium^23^. Following screening, the strains with better growth on the plate were selected and inoculated into 100 mL liquid fermentation medium with disposable inoculation ringthen subjected to 72 h of shaking cultivation at 30 °C and 150 rpm. Strains with robust cellulose-degrading ability were selected based on their CMCase activity levels.

#### Inoculum preparation

A single colony of the cultivated strains obtained from the initial samples was selected and inoculated into a large test tube containing 10 mL LB medium for incubation at 30 °C and 150 rpm for 24 h. Subsequently, 1 mL of the inoculum was transferred to 100 mL LB culture medium, maintaining the identical incubation conditions. The optical density (OD) of the bacterial solution measured at 600 nm^24^ using a spectrophotometer was 0.7–0.8 (OD_600_), resulting in a second-generation bacterial inoculum for subsequent experiments.

### Determination of cellulase activity

To assess the cellulase enzyme activity, 5 mL of the bacterial solution was drawn aseptically from the shaker flask and diluted 2X (diluted with medium).

CMCase activity was determined through the DNS method^25,26^: Briefly, 0.5 mL of the prepared enzyme was added to a 25 mL stoppered test tube, followed by the introduction of 1.5 mL of CMC Na solution and subsequent incubation in a water bath maintained at 50 °C for 30 min. After incubation, 1.5 mL of DNS reagent was added using a pipette, and the stoppered test tubes were then immersed in a boiling water bath for 5 min. Once cooled, the reaction solution was diluted with water to the 25 mL scale for recording the absorbance at 540 nm using an ultraviolet spectrophotometer^27^. One unit of CMCase activity (U) is defined as 1 mL of enzyme solution that catalyzes cellulose hydrolysis to produce 1 μg of glucose per minute.

The CMCase activity was calculated using the following equation:

$${\text{U}} = \frac{{{\text{P}} \times {\text{K}} \times 1000}}{{0.5 \times {\text{T}}}}$$

where U represents the enzyme activity; P refers to the glucose content obtained from the standard curve; K denotes the dilution factor of the enzyme solution; T indicates incubation time in a constant-temperature water bath.

A glucose standard curve^28,29^ was prepared using a working stock of 1.0 mg/mL glucose. To six graduated test tubes (25 mL) equipped with stoppers, glucose standard solution (1.0 mg/mL) was aliquoted as 0.0, 0.4, 0.8, 1.2, 1.6, and 2.0 mL, respectively, followed by the addition of distilled water inversely proportional to glucose solution (2.0, 1.6, 1.2, 0.8, 0.4, and 0.0 mL). Next, 1.5 mL of DNS reagent was added and the contents were thoroughly mixed before placing the tubes in the boiling water bath for 5 min. Post incubation, the test tubes were cooled down to room temperature by rinsing with running water. The solution was then adjusted to a consistent volume with distilled water and mixed vigorously to measure absorbance at 540 nm. The linear equation of the standard curve was established with glucose concentration as the independent variable and absorbance as the dependent variable.

### Growth curve of the bacteria isolate

To create a growth curve, the following steps were undertaken: the second-generation bacterial inoculum (see “Inoculum preparation” section) was transferred to NB liquid medium at a ratio of 1:100 and cultivated at 30 °C and 150 rpm. During the first 24 h post-inoculation, samples were harvested at every 2-h interval: 0, 2, 4, 6, 8, 10, 12, 14, 16, 18, 20, 22, and 24 h, resulting in 13 data points. Between 24 and 48 h, sampling was carried out every 4 h at 28, 32, 36, 40, 44, and 48 h, thus, creating 6 additional points. Immediately after each sampling, the samples were analyzed using a spectrophotometer at 600 nm. The sampling time was plotted on the horizontal coordinate and the corresponding OD_600_ on the vertical axis to generate the bacterial growth curve.

### Sequencing

The selected isolateswere submitted to Sango Biotech (Shanghai) Co. for 16S rDNA sequencing. Subsequently, the obtained 16S rDNA sequences were compared to the NCBI database employing BLAST to identify the bacterial genus of each strain.

### Single-factor experiments to optimize CMCase production conditions of the selected isolates

#### Effect of agitation speed on CMCase production

The experiments were performed with liquid fermentation medium at three different agitation speeds of 100 rpm, 150 rpm, and 200 rpm. Each fermentation involved 200 mL of liquid medium and 2 mL of strain inoculum, with incubation at 30 °C for 5 days. During incubation, samples were aliquoted at four different intervals on the 1st, 2nd, 3rd, and 5th days to determine the CMCase activity.

#### Effect of cultivation temperature on CMCase production

The experimental setup involved incubation at three distinct temperatures of 25 °C, 30 °C, and 37 °C while keeping other conditions akin to “Effect of agitation speed on CMCase production” section. The agitation speed was maintained at 150 rpm and during the 5-day incubation period, the CMCase activity was assessed at four sampling intervals on the 1st, 2nd, 3rd, and 5th days.

#### Influence of medium concentration on CMCase production

The base substrate concentration of the formulated liquid fermentation medium was set at 1C. Accordingly, the experimental substrate concentration was varied across three levels: 0.5C, 1C, and 2C (0.5C—means medium components concentration was reduced to half and 2C—double concentration), keeping the fermentation volume constant at 200 mL and the inoculum size at 2 mL. Five days of incubation occurred at 30 °C with an agitation speed of 150 rpm, and the CMCase activity was evaluated at different sampling intervals on the 1st, 2nd, 3rd, and 5th days.

#### Influence of inoculation volume on CMCase production

The experimental setup involved three different inoculum volume levels of 1 mL, 2 mL, and 5 mL, introduced to 200 mL of fermentation broth. Cultivation conditions were maintained at 30 °C and 150 rpm for a period of 5 days, and sampling was conducted on the 1st, 2nd, 3rd, and 5th days to determine the CMCase activity.

### RSM design to optime CMCase production conditions for isolate A49

Analysis of the single-factor experiment results identified three pivotal factors that strongly influenced the CMCase activity of cellulose-degrading isolate A49. A Box-Behnken-designed experiment, involving the 3 factors at multiple levels (Table 1), was executed to perform the RSM optimization of the CMCase production capacity of isolate A49 and validate the optimal CMCase production conditions. All experiments were carried out using the liquid fermentation medium in an agitation speed at 150 rpm. After a two-day cultivation period, samples were collected from the fermentation liquid of different flasks to quantify the CMCase activity.

**Table 1:** Level of Box-Behnken design factors.

Factor	Level		
A: Substrate concentration/C	1	2	3
B: Inoculation volume/mL	2	5	8
C: Temperature/°C	30	37	44

## Results

### Glucose standard curve

The glucose standard curve was established with a linear regression equation: y = 0.4812x + 0.0120, where R^2^ = 0.998. This equation served as the basis for calculating the amount of glucose produced after enzymatic action.

### Screening of strains

#### Primary screening

Viable isolates (designated as A) were retrieved from the culture medium and cultivated at 30 °C to observe their individual growth characteristics. Among these isolates, A3, A6, A16, A32, A33, A34, A35, A43, A44, A46, A50, A56, A57, A58, A60, A74, A75, and A76 were omitted from the study because of their insufficient growth rates and diminished activity, rendering them unsuitable for practical applications. The remaining isolated strains were inoculated onto Congo red medium, and the size of transparent zones formed by each strain was measured after ten days of cultivation at 30 °C. The results revealed that isolates A1, A11, A13, A24, A29, A49, A61, A69, and A70 exhibited ratios (D/d) greater than 2 (Table 2), indicating their proficiency in cellulose degradation. Conversely, isolates that failed to produce transparent zones were deemed incapable of degrading cellulose and were consequently excluded from further utilization.

**Table 2:** Size of transparent zone of different isolates.

Isolate	Diameter of colony(mm)	Diameter of transparent zone (mm)	Ratio (D/d)
A1	4.50	9.05	2.01
A11	4.09	8.36	2.05
A13	3.14	7.35	2.34
A24	2.68	8.90	3.32
A29	1.94	4.25	2.19
A49	2.65	9.73	3.67
A61	2.66	6.38	2.40
A69	1.92	4.12	2.15
A70	4.11	8.49	2.06

D, The diameter of transparent zone; d, The diameter of colony; Ratio = D/d.

#### Re-screening

**Figure 1:**
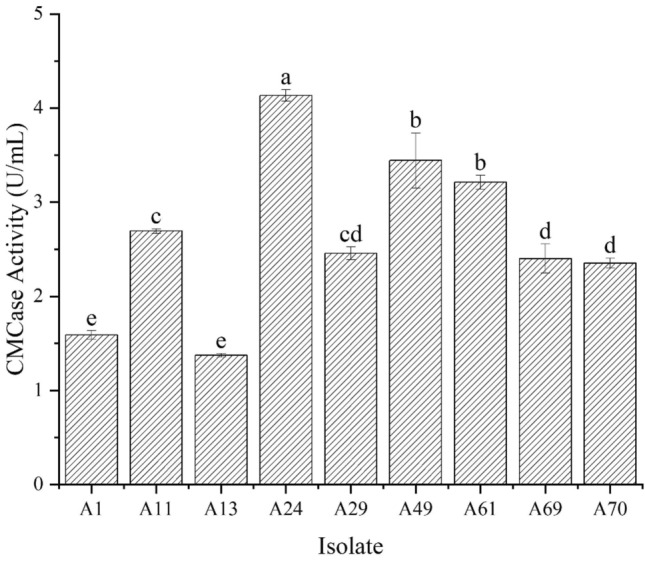
CMCase activity of different isolates.

Cultivation of the initially screened isolates with transparent zones on Congo red plates into fermentation broth for 72 h at 30 °C/150 rpm and subsequent determination of CMCase activity demonstrated enhanced activities of isolates A24 (4.13 U/mL), A49 (3.44 U/mL), and A61 (3.21 U/mL) compared to the other isolates (Fig. 1). This signifies that the three isolates A24, A49, and A61 possess an efficient CMCase secretion ability with potentially robust CMCase productivity.

#### Growth curve of the selected isolates

**Figure 2:**
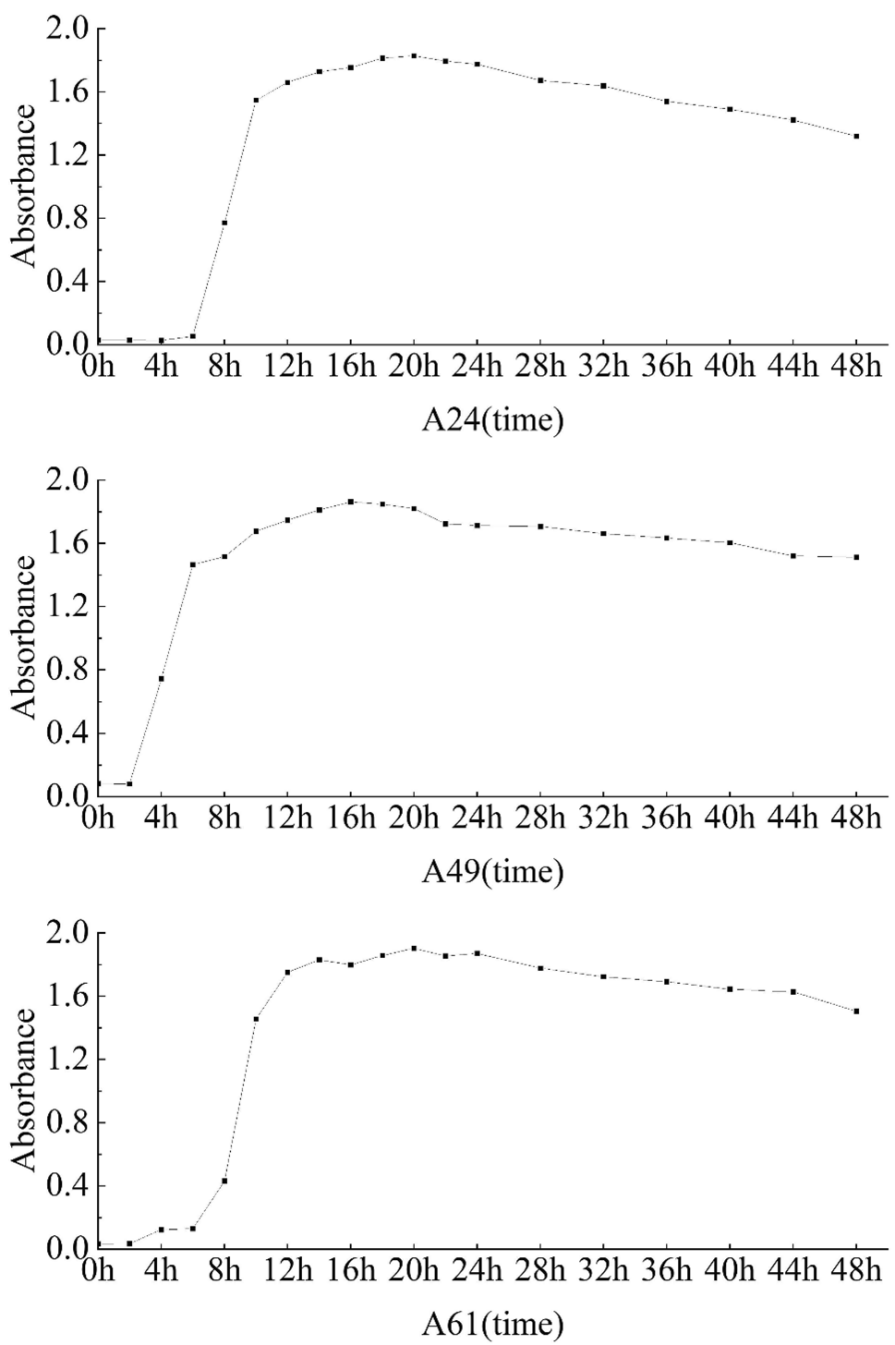
Growth curve of three isolates.

The growth characteristics of the three isolates (A24, A49, and A61) were evaluated in NB medium, as illustrated in Fig. 2. The growth curve of isolate A24 exhibited a logarithmic growth phase commencing at the 6th hour, followed by a growth plateau at the 14th hour, and ultimately the death phase at the 22nd hour. Likewise, isolate A61 entered the logarithmic growth phase at the 6th hour, progressed to the flat growth phase at the 12th hour, and eventually reached the dead phase at the 24th hour. Interestingly, isolate A49 displayed the logarithmic growth phase pattern as early as the 2nd hour, reached the slow growth phase at the 10th hour, and transitioned into the death phase at the 22nd hour.

#### Sequence analysis

**Figure 3:**
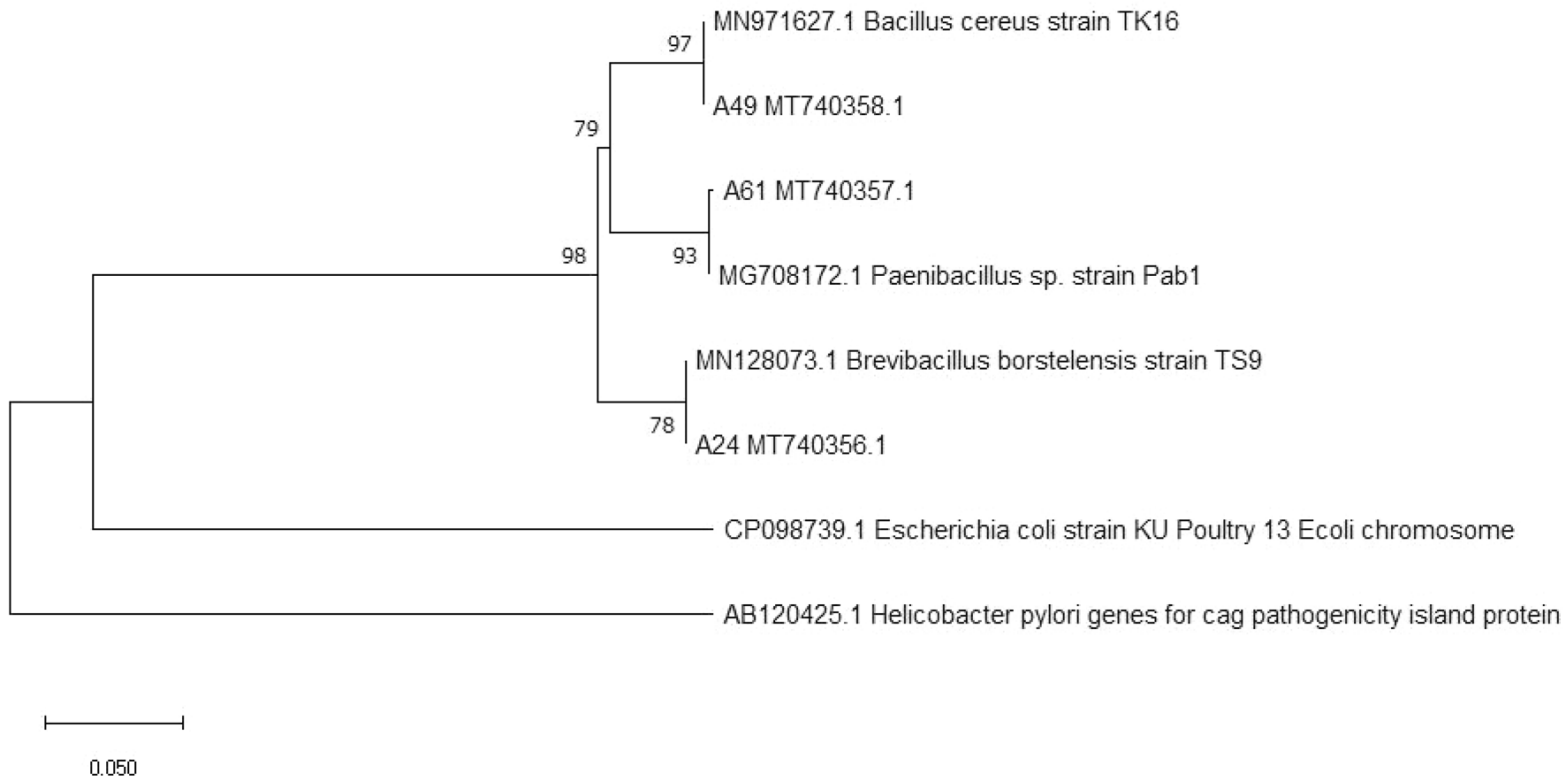
Phylogenetic tree of isolates A24, A49, and A61.

The sequencing results were analyzed by comparing them to the NCBI BLAST database, indicating a close homology of the isolates A24, A49, and A61, with *Brevibacillus borstensis* strain TS9, *Bacillus cereus* strain TK16, and *Paenibacillus* sp. strain Pab1, respectively (Table 3). Additionally, the obtained sequences were submitted to the Genbank database and assigned corresponding accession numbers as MT740356.1, MT740358.1, and MT740357.1. Furthermore, a phylogenetic tree was constructed using Molecular Evolutionary Genetics Analysis (MEGA)11 software, with a distance scale of 0.05, as depicted in Fig. 3. The Bootstrap value within the phylogenetic tree exceeded 70%, indicating the high credibility of the constructed tree. Based on these findings, it can be reasonably inferred that the isolates A24 (MT740356.1), A49 (MT740358.1), and A61 (MT740357.1) belong to *Brevibacillus borstelensis*, *Bacillus cereus*, and *Paenibacillus sp.* respectively.

**Table 3:** Sequence comparison of isolates.

Isolate	Strains with high homology	Similarity (%)
A24(MT740356.1)	*Brevibacillus borstelensis* strain TS9	100.00
A49(MT740358.1)	*Bacillus cereus* strain TK16	100.00
A61(MT740357.1)	*Paenibacillus* sp. strain Pab1	99.72

### Single-factor experiments for optimizing CMCase production by the selected isolates

#### Impact of varied agitation speed on CMCase production

**Figure 4:**
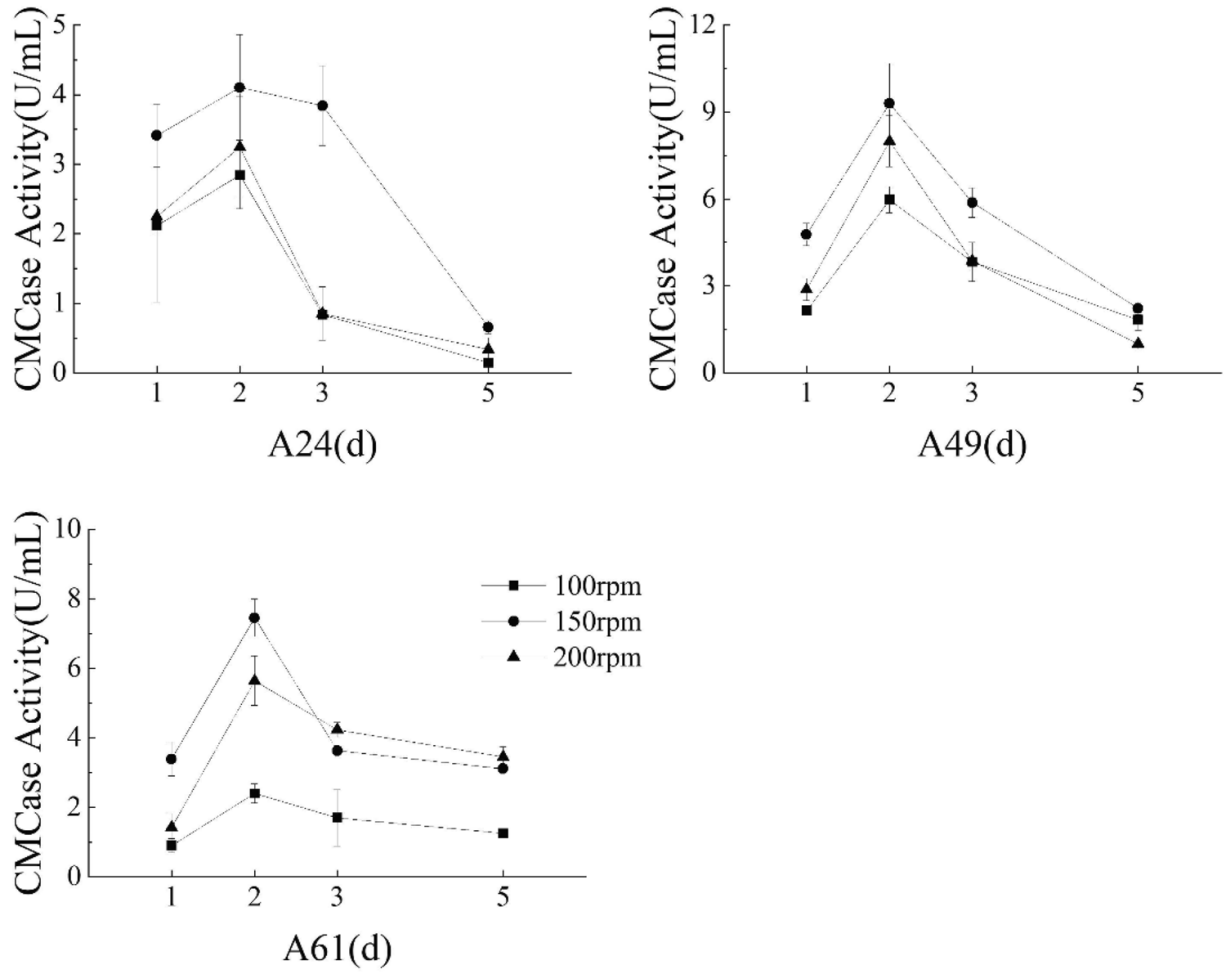
Different agitation speeds’ effects on CMCase activity of three isolates.

The CMCase activity displayed a pattern of an initial increase with gradual decline, reaching the maximum value on the second day and the lowest value on the fifth day (Fig. 4). Notably, maximum CMCase activity was achieved by the three isolates A24 (4.10 U/mL), A49 (9.30 U/mL), and A61 (7.46 U/mL) at 150 rpm agitation speed after two days of cultivation. Under these specific experimental conditions, the CMCase activity of isolate A49 surpassed that of both A61 and A24, demonstrating its superior efficacy in secreting CMCase.

During the initial two days of cultivation, the strain experienced logarithmic growth and remained in a stable phase, accompanied by a continuous increase in the total strain population and a concurrent rise in CMCase activity, with the maximum value achieved on the second day. However, the strain entered a declining phase starting from the third day, marked by a dramatic reduction in enzyme activity, which continued to decrease until reaching its lowest point on the fifth day. Importantly, the CMCase activity of the A49 at 150 rpm exceeded that of the other two (100 and 200 rpm) experimental conditions. Therefore, 150 rpm was observed as the optimal agitation speed for CMCase production by the present isolate.

#### Impact of different culture temperatures on CMCase production

**Figure 5:**
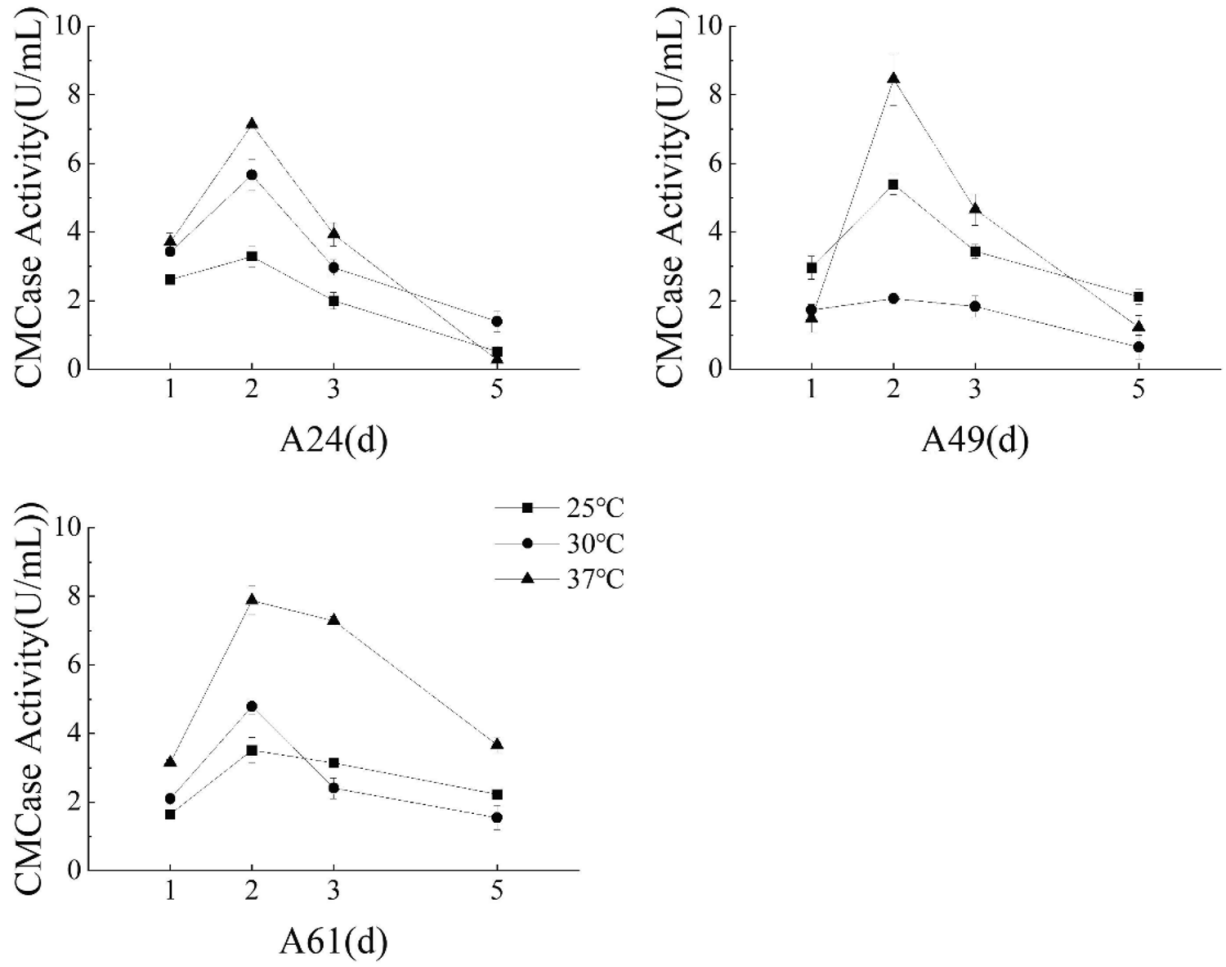
Different culture temperatures' effects on the CMCase activity of three isolates.

The CMCase activity of isolate A49 exhibited a consistent trend of initial increase followed by a subsequent decrease, with the peak achieved on the second day and the lowest activity recorded on the fifth day (Fig. 5). Moreover, the isolate A49 reached maximum activity of 8.46 U/mL at 37 °C after two days of incubation; the isolates A24 and A61 showed highest CMCase activity of 7.13 U/mL and 7.88 U/mL, respectively, under identical cultivation conditions. This highlights that the CMCase activity of isolate A49 was not notably distinct from that of isolates A24 and A61, indicating their comparable cellulase-secreting abilities. The growth characteristics were akin to the pattern observed during the optimization of shaker speed with a continuous increase in bacterial population and CMCase activity in the first two days and the lowest activity recorded on the fifth day with deteriorating bacterial growth. Remarkably, the CMCase activity of isolate A49 was higher at 37 °C than at 25 °C and 30 °C, aligning with the strain-specific culture conditions. Accordingly, a temperature of 37 °C could be recommended for regulating CMCase production in these isolates.

#### Influence of varying medium concentrations on CMCase production

**Figure 6:**
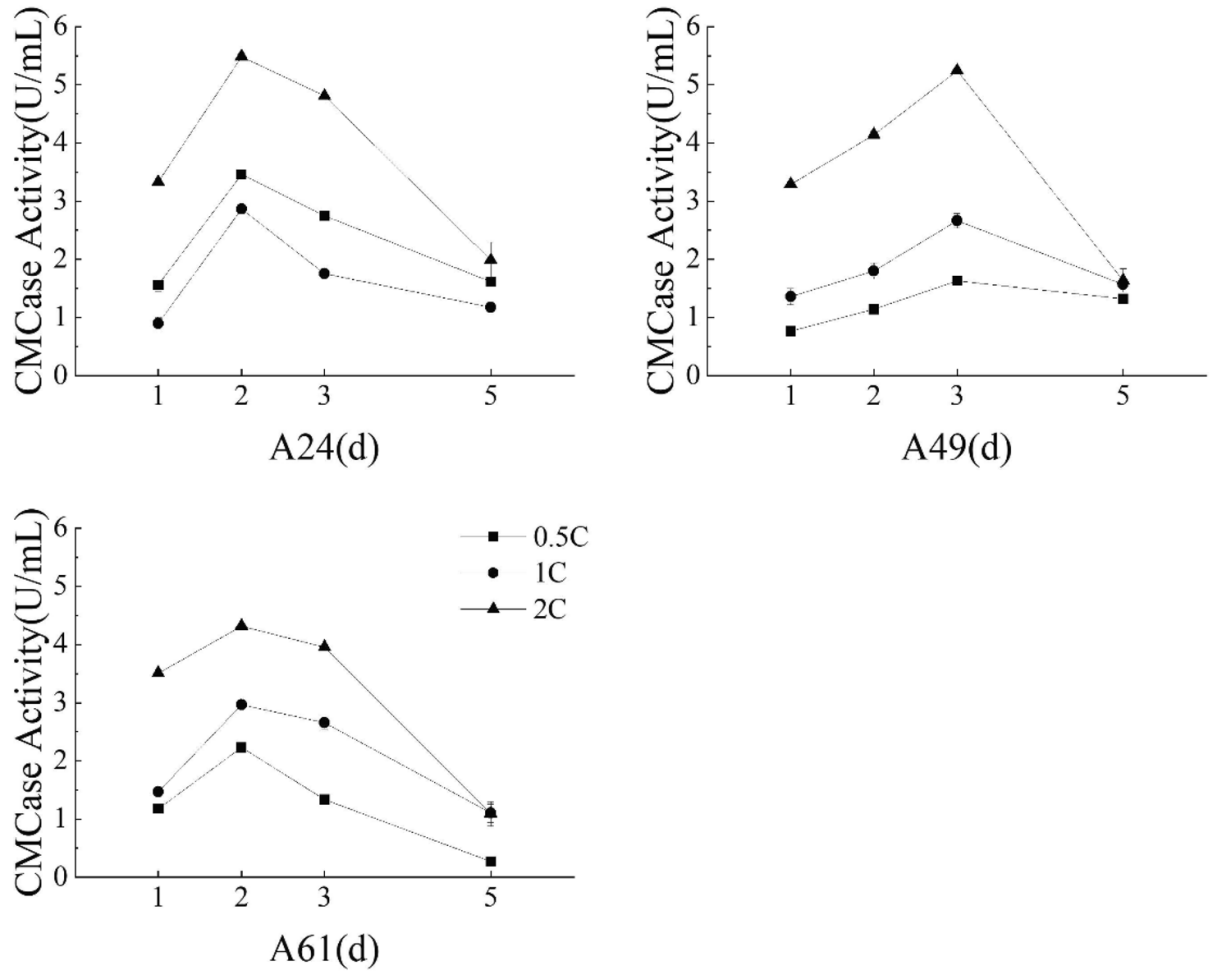
Different substrate concentrations' effects on CMCase activity of three isolates.

All three isolates showed a similar pattern of CMCase activity characterized first by an increase and then a gradual decline. Specifically, isolates A24 and A61 achieved their maximum activity value on the second day, which subsequently depreciated to the lowest point on the fifth day (Fig. 6). In contrast, isolate A49 attained its maximum activity on the third day. Cultivation using 2C substrate concentration for 2 days at 37 °C and 150 rpm resulted in CMCase activities of 5.48 U/mL and 4.32 U/mL for isolates A24 and A61, respectively; isolate A49 displayed a comparable activity of 5.24 U/mL after the three days of incubation at the same substrate concentration. The growth pattern of the three isolates was similar as observed during the optimization of cultivation temperature and shaker speed. Furthermore, the highest CMCase activity was obtained under 2C substrate concentration for all isolates after 2–3 days of incubation, followed by 1C and 0.5C. Consequently, 2C substrate concentration with the highest CMCase activity emerged as the effective experimental condition for optimal CMCase production by the isolates.

#### Effect of different inoculum volume on CMCase production

**Figure 7:**
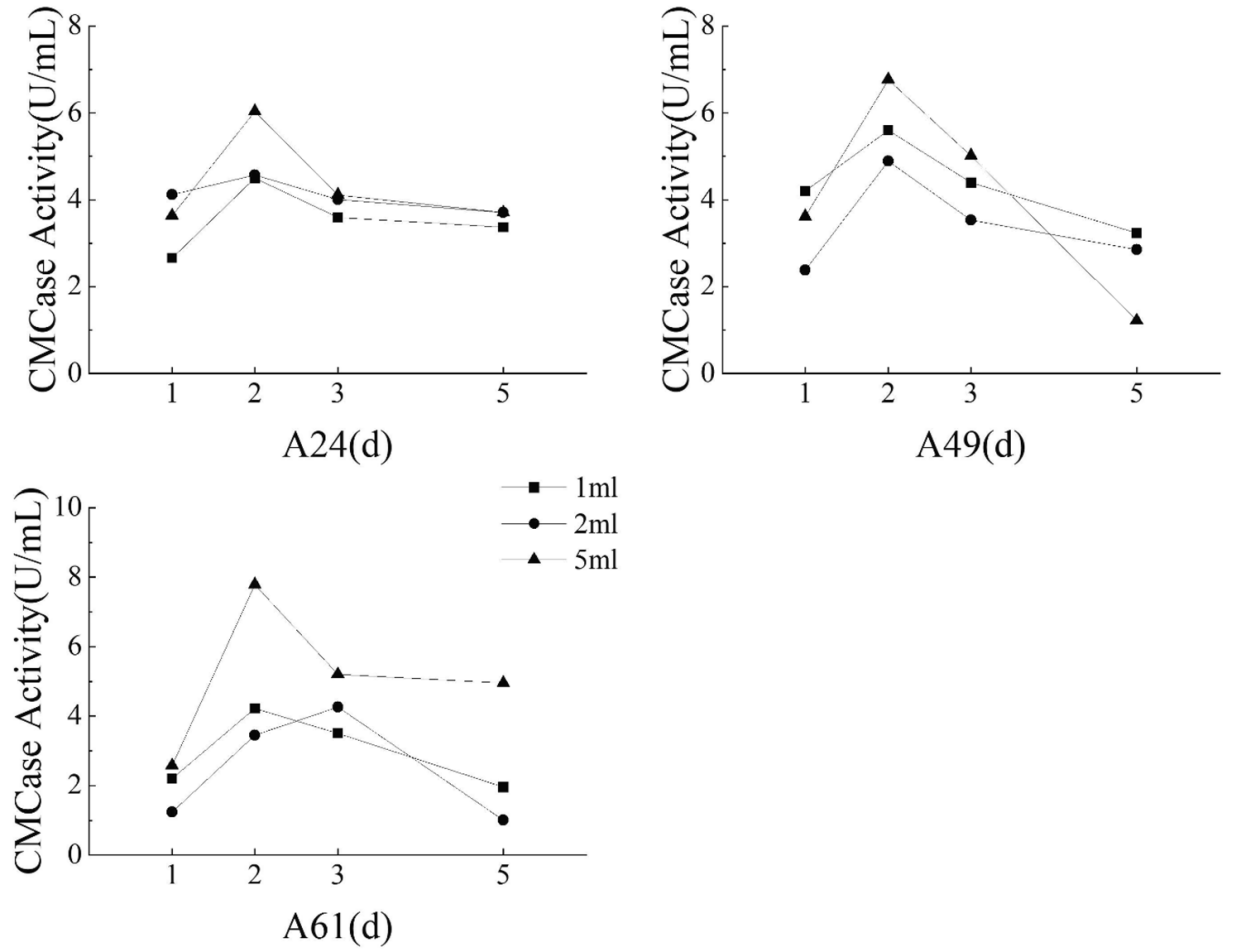
Different bacterial load’s effects on the CMCase activity of three isolates.

The CMCase activity of isolates A24, A49, and A61 reached the maximum value of 6.04 U/mL, 6.77 U/mL, and 7.80 U/mL, respectively, following a two-day incubation with 5 mL of inoculum (Fig. 7). Notably, the difference in the CMCase activity was insignificant among the three isolates, suggesting comparable secretion of cellulose-degrading enzymes. Growth characteristics were identical to those observed for other experimental factors, including shaker speed, cultivation temperature, and medium concentration. The CMCase activity was considerably higher using 5 mL inoculum compared to 1 mL and 2 mL of inocula, which exhibited no considerable difference. Accordingly, 5 mL inoculum could be considered optimal for enhancing CMCase production by these isolates.

### Optimization of CMCase production by isolate A49 employing RSM

#### Model establishment and significance analysis

Considering the remarkable similarity in CMCase production capacity among the three isolates under varying culture conditions, it was deemed feasible to optimize the conditions based on the CMCase production parameters of one isolate. According to the cultivation conditions, growth curve, and single-factor experiment results, isolate A49 was selected for RSM studies to optimize CMCase production conditions. The Box-Behnken design involved three key factors dramatically influencing the CMCase activity of isolate A49: temperature (30 °C, 37 °C, and 44 °C), inoculum volume (2 mL, 5 mL, and 8 mL), and substrate concentration of CMCase-producing medium (1C, 2C, and 3C). During the experimentation, the CMCase activity served as the response parameter (Table 4). Regression analysis was performed using Design Expert 10.0.3 to derive the quadratic multinomial regression equation as presented below^30^:

$$\begin{aligned} & {\text{CMCase}}\;{\text{activity}} = + 16.17 + 1.57{\text{A}} - 0.20{\text{B}} - 1.18{\text{C}} - 0.56{\text{AB}} \\ & \quad - 0.93{\text{AC}} + 0.62{\text{BC}} - 4.10A^{2} - 6.86B^{2} - 5.42{\text{C}}^{2} \\ \end{aligned}$$

**Table 4:** Box-Behnken design portfolio and results.

Serial number	Substrate concentration (C)	Inoculum volume (mL)	Temperature (°C)	CMCase activity (U/mL)
1	1	2	37	4.63
2	3	2	37	6.53
3	1	8	37	5.03
4	3	8	37	4.69
5	1	5	30	4.66
6	3	5	30	12.04
7	1	5	44	3.14
8	3	5	44	6.79
9	2	2	30	5.22
10	2	8	30	3.89
11	2	2	44	2.66
12	2	8	44	3.82
13	2	5	37	14.71
14	2	5	37	17.63
15	2	5	37	16.17
16	2	5	37	15.42
17	2	5	37	16.92

CMCase activity calculated based on experimental results.

The model constructed using CMCase activity as the evaluation index (Table 5) exhibited an F value of 19.42 indicating that the model was significant. A “Prob > F” value less than 0.0500 implied the significance of the model with enhanced reliability. The lack-of-fit with a *P* value of 0.1365 (> 0.05) indicated no loss-of-fit factor, which could be deduced from the regression equation. Furthermore, the selected model displayed an adequate fitting, with R^2^ = 0.9615. While monitoring interactions among the factors, A^2^, B^2^, and C^2^ demonstrated highly significant effects (*P* < 0.01), whereas A showed significant effects (*P* < 0.05). Besides, AB, AC, and BC interactions were more significant than 0.05, suggesting relatively weak interactions. Factors affecting CMCase activity were ranked from the most influential to the least: A (substrate concentration) > C (temperature) > B (inoculum volume).

**Table 5:** Box-Behnken test results of isolates.

Source	sum of squares	Df	Mean square	*F* value	*P* value Prob > F	Significant
Model	472.94	9	52.55	19.42	0.0004	Significant
A-substrate concentration	19.81	1	19.81	7.32	0.0304	*
B-inoculum volume	0.32	1	0.32	0.12	0.7395	
C-temperature	11.05	1	11.05	4.08	0.0831	
AB	1.25	1	1.25	0.46	0.5178	
AC	3.48	1	3.48	1.29	0.2942	
BC	1.55	1	1.55	0.57	0.4738	
A^2^	70.61	1	70.61	26.09	0.0014	**
B^2^	197.86	1	197.86	73.12	< 0.0001	**
C^2^	123.58	1	123.58	45.67	0.0003	**
Residual	18.94	7	2.71			
Lack of fit	13.55	3	4.52	3.35	0.1365	Not significant
Pure error	5.39	4	1.35			
Cor total	491.88	16				

*P < 0.05, significant difference; **P < 0.01, extremely significant difference.

#### RSM analysis

**Figure 8:**
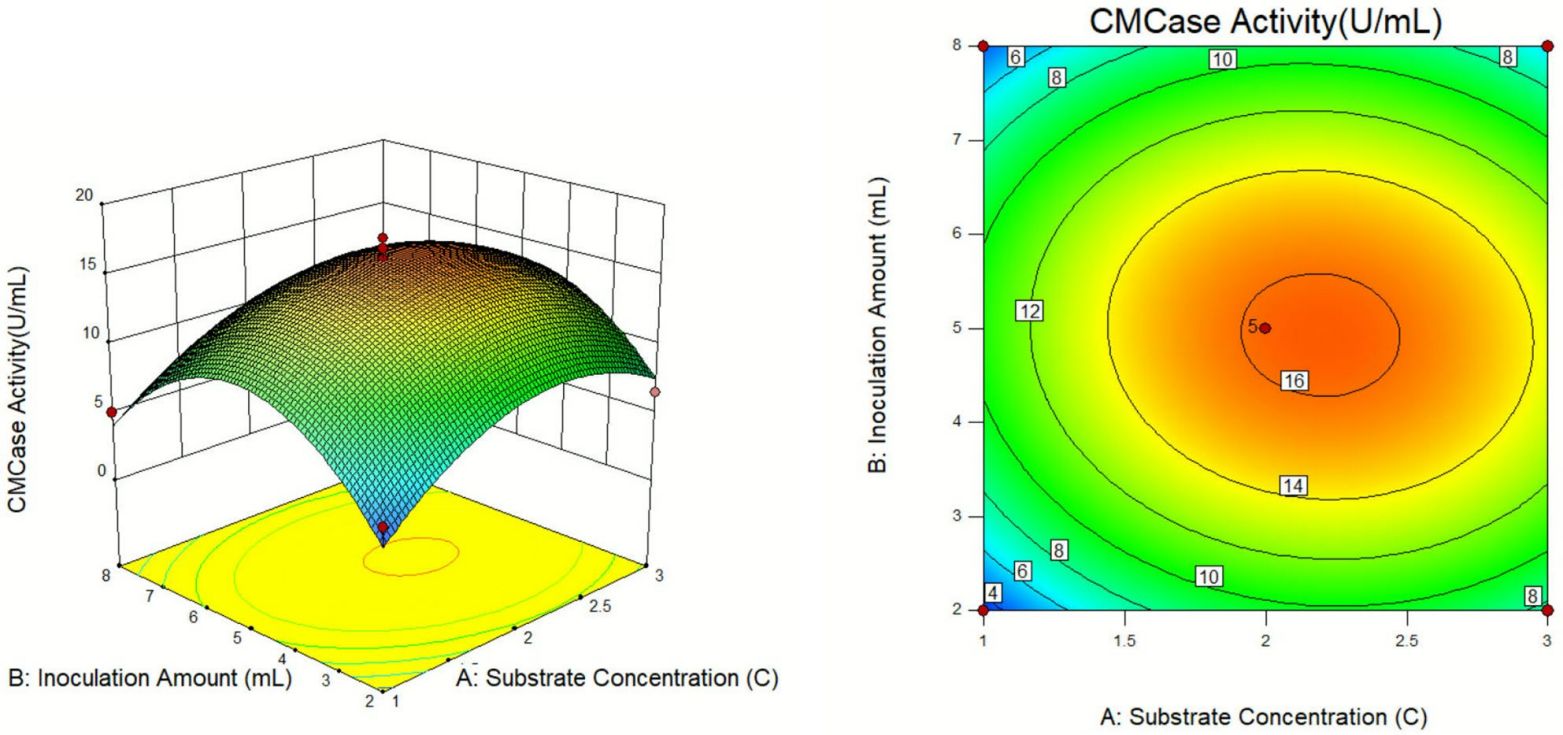
Response surface diagram and contour map of A and B interaction's effects on the CMCase activity.

**Figure 9:**
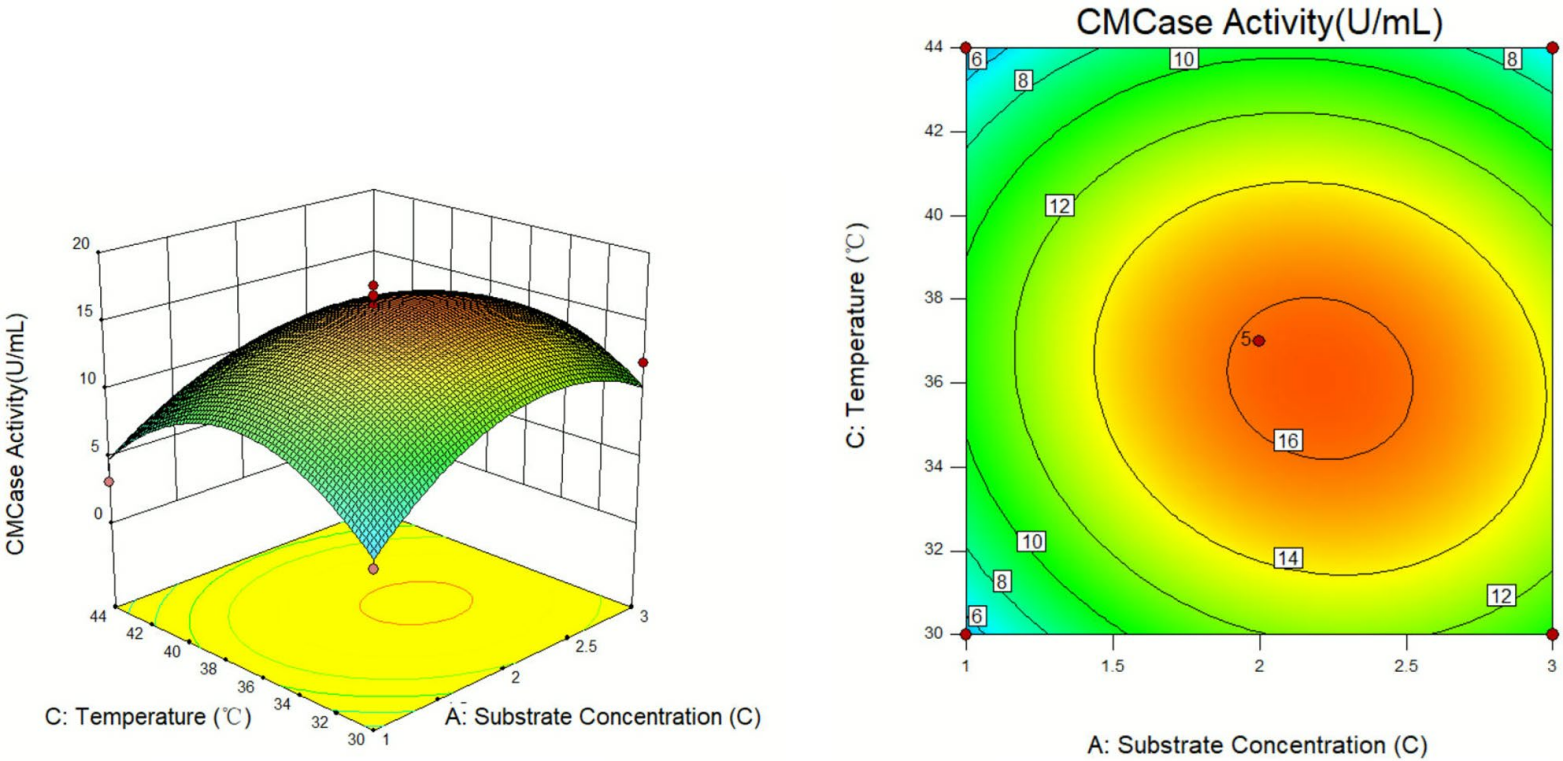
Response surface diagram and contour map of A and C interaction's effects on the CMCase activity.

**Figure 10:**
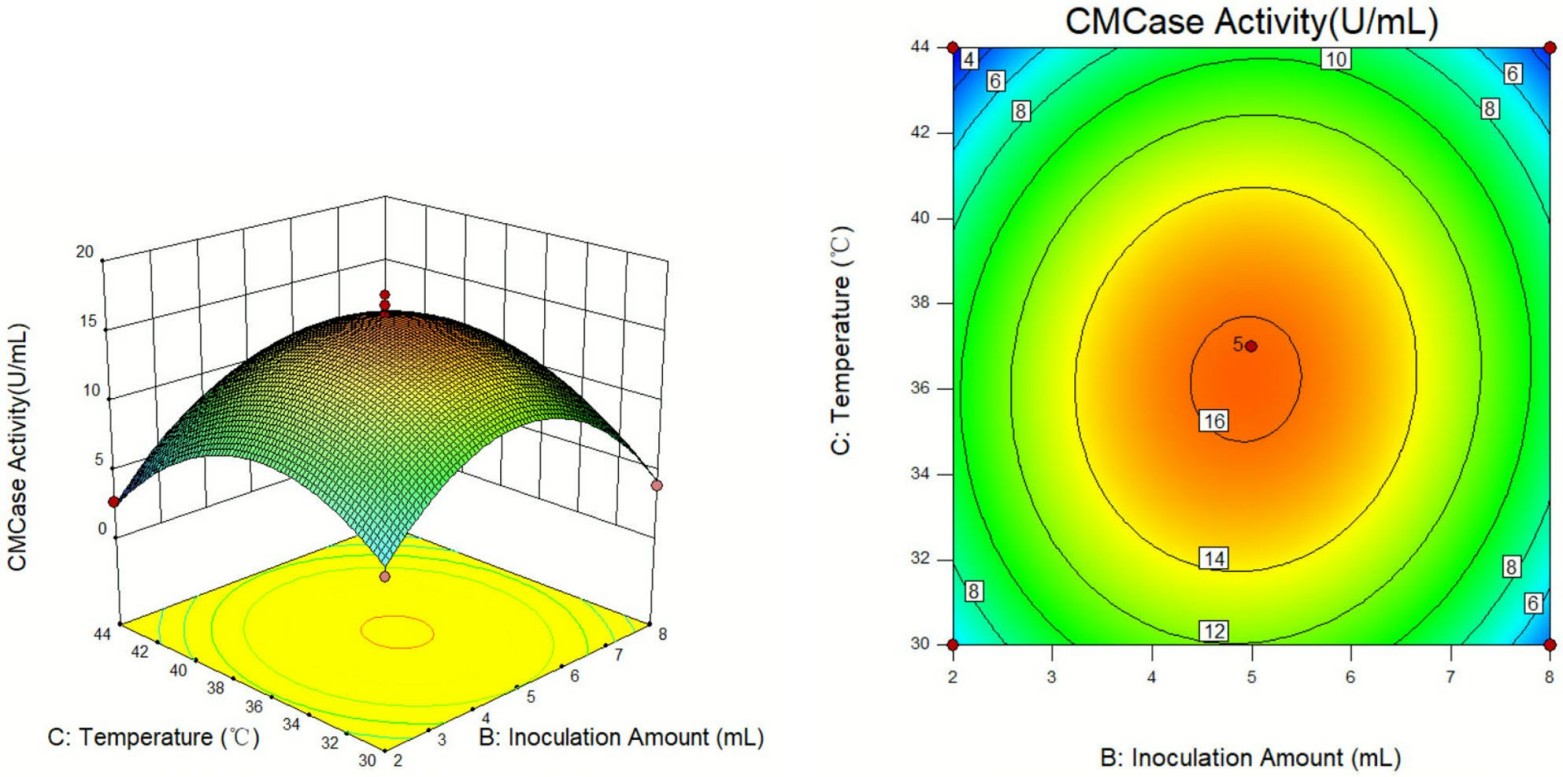
Response surface diagram and contour map of B and C interaction's effects on the CMCase activity.

The response surface curves and contour diagrams illustrating the interaction among the three factors viz. substrate concentration, inoculation volume, and cultivation temperature influencing CMCase activity are presented in Figs. 8, 9 and 10.

Analysis of the response surface curves revealed that sharper trends signified a more pronounced impact on the experimental outcome, while gentle trending indicated a lesser influence on the results. Regarding the contour maps, a curve with a steeper slope highlights crucial interaction between the two factors. In contrast, a shallow slope curve suggests a minor interaction of the two factors^31^. As shown in Fig. 8, at a constant temperature, the CMCase activity exhibited a pattern of both increase and decrease with the increase in substrate concentration and bacterial load. Maximum activity was achieved at a substrate concentration of 2.1–2.3C and an inoculation volume of 4.8–5.2 mL. Similarly, as shown in Fig. 9, the CMCase activity displayed a trend of the initial rise and subsequent decline at a fixed inoculum volume with increasing substrate concentration and temperature. The highest CMCase activity was attained under the substrate concentration range of 2.1–2.3C and temperature range of 35.5–37.0 °C. Figure 10 shown the effect of inoculum volume and temperature on CMCase activity when substrate concentration was constant. The highest CMCase activity occurred when the inoculum volume was 4.8–5.2 ml and the temperature was 35.5–37.0 °C. The response surface analysis demonstrated that the factors influencing cellulase enzyme activity could be ranked from most to least impactful: A (substrate concentration) > C (temperature) > B (inoculum volume).

Design Expert 10.0.3 software analysis revealed that the optimal enzyme production conditions for isolateA49 were: substrate concentration of 2.21C, inoculum volume of 4.91 mL, and temperature of 36.11 °C, with the predicted CMCase activity value of 16.41 U/mL^32^. To confirm the reliability of response surface optimization, the predicted conditions were adopted for practical experimentation. Considering the actual operating conditions in the experiment, the optimized conditions were modified to a substrate concentration of 2.2C, an inoculum volume of 4.9 mL, and a temperature of 36 °C. Under these modified conditions, the average CMCase activity across three experiments was determined to be 15.63 U/mL, which was parallelly close to the predicted value generated by the model. These findings support the feasibility of the model for optimizing CMCase production conditions for cellulose-degrading bacteria and underscore its specific application potential.

## Discussion

Currently, the majority of research concerning cellulose-degrading organisms has been centered around fungal strains, with limited focus on cellulose-decomposing bacteria^31,33^. However, bacterial strains capable of degrading cellulose exhibit a broad spectrum of adaptability to complex and dynamic environmental conditions compared to fungal strains. Moreover, vigorous growth rates of bacteria enable prolific production of cellulase, an advantage that slow-growing fungal organisms cannot rival^34^. In this study, three bacterial cellulose-degrading isolates, namely A24, A49, and A61, were screened and isolated from mixed soil samples. The growth curve analysis of these isolates demonstrated their remarkable ability to proliferate rapidly, providing conducive conditions for robust cellulase production. In another study, an F3 strain belonging to *Bacillus amyloliquefaciens*, isolated from fermented soybean products, displayed a cellulase activity of 21.14 U/mL under optimal cultivation conditions (37 °C, pH 7.0, and 6% inoculum volume)^35^. Likewise, the CMCase activity of *Bacillus subtilis* N5 identified from cow manure compost reached 189.27 U/mL under the most favorable culture conditions (pH 5.0, and 40 °C)^36^. Similarly, in this study, the culture conditions of the isolates were optimized through changes in agitation speed, cultivation temperature, medium concentration and inoculation volume. Under optimized conditions, the isolates grew vigorously and secreted more CMCase, which greatly improved the CMCase activity of the isolates.

A previous report by Li et al.^37^ employed a similar experimental approach to screen a cellulose degrading bacterium C-19. Subsequent investigations unveiled that the strain exerted a biological strengthening effect on the cellulose degradation system of fly maggot biotransformation and silkworm sand, offering valuable insights for strain cultivation and development. Furthermore, the enzyme production conditions of *Bacillus velezensis* strain D103 were optimized to obtain high cellulase activity^38^. Besides, numerous studies have documented the advantage of utilizing *Bacillus* strains in the secretion of various proteins. Considering the many advantages of *Bacillus*, we chose *Bacillus* cellulose-degrading bacteria as the research target. In this study, analysis of single-factor outcomes indicated that the *Bacillus cereus* A49 exhibited superior CMCase production ability compared to the other two isolates. Therefore, the isolate A49was selected for response surface optimization studies. RSM is a well-established and versatile scientific approach with extensive applicability across various domains^39^. For example, (Mustefa et al.^40^) effectively adopted RSM to optimize protease production from *Aspergillus*, considering the three key parameters: temperature, pH, and sucrose concentration. Another study (Udume et al.^41^) employed RSM to enhance the lignocellulolytic degradation in water hyacinth, consequently improving the cellulose degradation process and soil fertility through optimized composting products. These studies have used RSM, which has an important role in promoting experimental research. Similarly, in the study of RSM optimizing the culture conditions of cellulose-degrading bacteria, (Liu et al.^39^) through the single factor experiment and the joint application of RSM, concluded that the optimal culture conditions of CE41 were the optimal temperature 38.67 °C, pH 6.93, and inoculation volume 6.14 mL. In this study, the same experimental method was used to optimize the conditions of CMCase production by cellulose degrading bacteria A49, resulting in a marked improvement in production capacity and CMCase activity. The highest CMCase activity was attained under the substrate concentration of 2.2C, inoculum volume of 4.9 ml and temperature of 36 °C. Under optimal conditions, the isolate A49 could potentially mitigate the low utilization of cellulose in agricultural and sideline products to effectively minimize the wastage of agricultural resources^42^. The biodegradation of cellulose is recognized as an economical and green treatment method. In this study, the screened cellulose degrading bacteria can be directly used for waste crop composting, effectively degrading cellulose in compost and reducing resource waste. The produced cellulase has high activity and can be used as an excellent strain for producing cellulase. This carries substantial implications for the realistic exploitation and utilization of cellulose resources, thus bolstering the prospective application of isolate A49 in the cellulose industry. This study only analyzed the influence of several single factors on the culture conditions of cellulose-degrading bacteria, which makes this study have certain limitations. Future research should focus on constructing recombinant gene expression system using genetic engineering methods. It can break through the growth defects of the strain itself, maximize the enzyme production capacity of the strain, and make rapid and targeted modifications to the fermentation strain to express the desired traits. This will provide a new technical way to promote the industrial application of cellulase.

## Conclusion

This study has demonstrated the effective cellulose degradation capabilities of the three selected isolates, designated A24, A49, and A61. Single-factor optimization provided favorable culture conditions, including agitation speed of 150 rpm, 37 °C cultivation temperature, twice the amount of basic substrate addition, and 5 mL inoculum volume, to attain maximum CMCase activity by the isolates. Additionally, response surface optimization focusing on the CMCase production conditions for the isolateA49 yielded substantial improvement in the CMCase production and activity. Based to the optimization studies, the average CMCase activity of the isolate A49 reached 15.63 U/mL. Hence, the isolatesscreened in this study can provide excellent strain resources for the development and utilization of cellulose degrading strains and serve as a valuable reference for the screening and cultivation of similar strains in future research endeavors.

## Data Availability

All data generated and analyzed during this study are included in this published article and sequences were deposited in NCBI-GenBank with accession numbers, MT740356.1, MT740358.1, MT740357.1, MN971627.1, MG708172.1, MN128073.1, CP098739.1, AB120425.1.
